# Stopover use of a large estuarine wetland by dunlins during spring and autumn migrations: Linking local refuelling conditions to migratory strategies

**DOI:** 10.1371/journal.pone.0263031

**Published:** 2022-01-25

**Authors:** Teresa Catry, José Pedro Granadeiro, Jorge Sánchez Gutiérrez, Edna Correia

**Affiliations:** 1 Departamento de Biologia Animal, Centro de Estudos do Ambiente e do Mar (CESAM), Faculdade de Ciências, Universidade de Lisboa, Lisbon, Campo Grande, Portugal; 2 Department of Anatomy, Cell Biology and Zoology, Conservation Biology Research Group, Faculty of Sciences, University of Extremadura, Badajoz, Spain; 3 Ecology in the Anthropocene, Associated Unit CSIC-UEX, Faculty of Sciences, University of Extremadura, Badajoz, Spain; University of Veterinary Medicine Vienna: Veterinarmedizinische Universitat Wien, AUSTRIA

## Abstract

Migratory strategies dictate stopover ecology, particularly concerning decisions of when, where and how long to stop, and what to do at stationary periods. In birds, individuals stop primarily to replenish energy stores, although the functions of stopover events vary among and within species, particularly between pre- and post-breeding seasons. Here, we combined plasma metabolite levels and haematological parameters to compare refuelling rates and physiological state within (early, mid, late) and between (spring, autumn) migratory periods, aiming to identify potentially different migratory strategies in a shorebird, the dunlin *Calidris alpina*, using a key stopover site in Iberia. Plasma triglycerides and β-hydroxybutyrate concentrations did not differ between seasons, and small differences were found in haematological profiles (higher haemoglobin and hematocrit levels in spring). Similar refuelling rates and physiological status suggests a single migratory strategy in spring and autumn. During both seasons, dunlins arrive at the Tagus estuary with medium-to-high fuel loads, indicating they do not engage in prolonged fuelling. This agrees with a skipping migratory strategy, where birds fly short-to-medium distances while fuelling at moderate rates along a network of sites. Although we may expect late spring migrants to experience stronger pressures to optimally schedule migratory events, we found no significant differences in physiological profiles among early, mid and late migrants. Unexpectedly, such differences were found in autumn: early birds showed the highest triglycerides and haemoglobin levels and lowest β-hydroxybutyrate concentrations. These results denote enhanced refuelling rates and blood oxygen-carrying capacity in early autumn migrants, which is typical of jumpers, i.e., birds travelling with larger fuel loads and performing fewer stops. Our study adds substantially to previous knowledge of stopover ecology in migratory shorebirds in the East Atlantic Flyway. Importantly, it indicates that the Tagus estuary is a high-quality stopover site for intermediate fuelling. Yet, understanding non-fuelling stopping functions is needed to ultimately inform conservation planning.

## Introduction

Seasonal migration has evolved to maximize the survival and reproductive output of animals in response to seasonal or geographic variation of resources, habitats, predators, diseases or competitors [1, 2]. Animals have developed a wide range of strategies to tackle the challenges faced during their journeys mostly under the selective forces of time, energy and predation minimization [3]. Optimal migration theory provides the framework to understand migratory patterns in a large number of bird species [e.g. 4–6]. Time-minimizers reduce the total time of migration by travelling with larger fuel loads which allows them to make fewer (but usually longer) stopovers while *en route* [3, 7]. In contrast, energy-minimizers reduce the total energy cost of migration by making more (but often shorter) refuelling stops during their journeys, thus carrying smaller fuel loads during flights [3, 7].

Migratory strategies vary widely among and within species and are generally shaped by total migration distance [8] and the selective pressures faced during different seasons, which are mediated by life-cycle stages and environmental conditions [9–12]. Birds embarking on their pre-breeding migration (hereafter spring migration, for boreal temperate breeders) tend to follow a time-minimization strategy [12]. Such strategy arises as a response towards the urgency of an early arrival at the breeding grounds to defend a territory [13] and ensure that breeding can be quickly initiated, thus matching the narrow window of resource availability and avoiding phenological mismatches [14, 15]. Since time constrains decrease during the post-breeding migration (hereafter autumn migration) and, simultaneously, environmental conditions such as weather and food availability improve, birds more often adopt an energy-minimization strategy [5, 12]. While this pattern for a faster migration in spring compared to autumn seems to apply for a large number of species (review in [12]), a growing number of exceptions is being revealed among arctic and subarctic birds [16–21]. A rapid autumn migration can be advantageous if birds suspend or postpone moult until they reach their non-breeding grounds, thus avoiding a challenging overlap between moult and migration [19]. Migratory strategies may also vary at the individual level within the same season, which is mainly driven by different decisions on departure time [22, 23]. Particularly during spring, late migrants are more time-constrained and thus more likely to follow a stricter time-minimization strategy in order to optimally schedule migration events.

Sites along migratory routes where birds spend stationary periods have been classified as either “stopover” or “staging” sites, with the former acting more as a “resting” spot, where birds stay for short periods, and the latter being used for long refuelling periods [24, 25]. However, the ability of birds to change migratory behaviour between and within seasons may deliver a variable role to a same stopover site according to individual’s internal state and the external environment [26]. Therefore, a single site can be differently used by the same population in different seasons, or by different individuals within the same season, according to date and their tightness to the migratory calendar.

Assessing the physiological profile of individuals at stopover areas is widely used method to obtain reliable information on key parameters of migration, while also providing information concerning the metabolic mechanisms involved in migratory decisions. Refuelling rates have been shown to be strongly linked to stopover duration, departure fuel load and overall migration speed [27–29]. Rates of fuel deposition (and catabolism) can be estimated by measuring the plasma metabolite profile of an individual sampled in a single capture event [30–32]. Notably, triglycerides are the major form of stored fuel in birds, and plasma concentrations of triglycerides rise during fat deposition (high refuelling rates) as they are transported to adipose tissue as lipoproteins [31, 33]. Conversely, during fasting and mass loss, i.e. when energy demand exceeds intake, glycerol concentrations increase, as stored lipids in the form of triglycerides are mobilized from adipose tissue and converted into glycerol and fatty acids [33]; at the same time β-hydroxybutyrate is produced by the liver as an alternative fuel source [31, 33]. Although lipids are the primary fuel for migratory flights, proteins are also a fuel source. Plasma concentrations of uric acid have been shown to increase when protein catabolism occurs and may, therefore, be higher in individuals arriving from long flights [32]. Besides, haematological parameters (e.g. leucocyte profiles, haematocrit, haemoglobin concentrations) provide some indication on the physiological status of migrating birds [34–36]. Haematocrit and haemoglobin, in particular, have been shown to increase in migratory birds during refuelling periods in order to elevate their oxygen-carrying capacity of blood prior to departure on a migratory flight [34, 37]. Previous studies also suggested that species following an energy-minimization strategy have lower blood oxygen-carrying capacity than time-selected species which often perform longer endurance flights [36]. Leucocyte profiles have been linked to energetic stress and immunocompetence in migrating birds [35]. For instance, high heterophil/lymphocyte ratios and low numbers of lymphocytes and monocytes were recorded in red knots (*Calidris canutus*) arriving at their southern non-breeding site after an extremely long migratory flight, suggesting that birds were energetically stressed and possibly immunocompromised [35]. Whether such physiological differences can be associated to migratory strategies at the population or individual level is unknown.

Shorebirds are remarkable migrants, and their migratory strategies are commonly pooled in three categories along a time-energy minimization continuum: jumping, skipping and hopping [19, 24, 38]. In this study, we compared the physiological profiles of dunlins (*Calidris alpina*) using a key wetland for migratory shorebirds in the East Atlantic Flyway (EAF), within (early, mid and late) and between (spring and autumn) migratory periods. In doing so, we linked physiological profiles to migratory strategies and local resource availability, aiming to understand whether dunlins use different migratory strategies according to their life cycle and migratory calendar. The Tagus estuary holds internationally important numbers of non-breeding (wintering) birds but also acts as a strategic stopover and/or staging site for many species during the pre- and post-breeding migration [39–41]. Here, we combined plasma metabolite levels and haematological parameters to compare the refuelling rates and physiological state of migrating dunlins. Additionally, as a proxy for site quality, we assessed and compared food availability and feeding performance of migrants in spring and autumn. We hypothesized that if dunlins perform a hopping migratory strategy and are more time-constrained during spring migration, they will show lower refuelling rates (lower triglycerides and higher β-hydroxybutyrate and glycerol concentrations) and higher blood oxygen-carrying capacity compared to autumn migrants, with the same pattern applying to late migrants, particularly during spring.

## Materials and methods

### Study area and study species

This study was carried out in the Tagus estuary (38⁰ 45’ N, 09⁰ 01’ W, Portugal), one of the largest estuarine wetlands in Europe and the second most important area for shorebirds in the Iberian Peninsula [42]. The estuary holds non-breeding (overwintering)and passage (both spring and autumn) populations of dunlins, which overlap over short periods. The nominate subspecies, *C*. *a*. *alpina*, breeds from northern Scandinavia to Western Siberia, and comprises the large majority of the Tagus’s overwintering population, whereas the subspecies *C*. *a*. *shinzii* breeds mainly in Iceland and winters in Mauritania and comprises the vast majority (c. 97%) of the population that stopover at the Tagus estuary during migratory periods [42–44]. During spring, ca. 30,000 dunlins use the Tagus estuary as a stopover site for an average period of 7.5 days [40]. Population size and stopover length of autumn migrants is unknown, but numbers recorded in a long-term monitoring program based on monthly high-tide counts suggest that similar populations may be involved in spring and autumn migration periods [41].

### Capture and sampling of migratory dunlins

Dunlins were captured with mist-nets at a high-tide roost throughout spring (five capture events between 7^th^ April and 17^th^ May) and autumn (three capture events between 18^th^ July and 16^th^ August) migration periods in 2019. The very first spring migrants are known to arrive at the Tagus estuary by late March [43, 44] and the later individuals arrive around mid-May [40, 41]. In autumn, migrant dunlins arrive in the second half of July and the numbers peak by late August [41], but the exact end of the passage period is unknown and by late August some of the overwintering birds also start arriving (authors pers. obs.). For this reason, we finished the autumn sampling period in mid-August.

All birds were ringed and had their body mass (to the nearest 0.1 g), wing length (maximum chord, to the nearest mm), fat score (0–8 scale, following [45]) and presence of wing moult recorded. Age was determined following the criteria described by Prater et al. [46], and only adult birds were included in the analysis. A blood sample (0.5 ml) was collected from the right jugular vein and several subsamples were taken for different analyses. One drop of blood was stored in absolute ethanol for molecular sexing, another drop was used to determine haemoglobin concentrations using a portable HemoCue Hb 201 (HemoCue Hb, Ängelholm, Sweden), and a third drop was used for a blood smear using the 2-slide wedge technique [47]. Blood samples were kept refrigerated in a portable cooler box before being centrifuged for 10 min at 3,400 rpm within a maximum of 4 hrs of collection to separate red blood cells (RBC) and plasma. A subsample of approximately 75 μL was used to determine haematocrit (in loco). The mean cell haemoglobin concentration (MCHC), calculated as the ratio between haemoglobin and haematocrit, was used as a measure of haemoglobin content relative to cellular blood fraction [34]. The remaining blood (plasma and RBC) was transported in a portable cooler to the laboratory, where it was stored at—20°C until plasma metabolite and stable isotope analysis (SIA) were performed. SIA was conducted in plasma and RBC and used to estimate the arrival date at the Tagus estuary of spring migrants and to infer previous stopover sites in autumn migrants (see “Data analysis”). Time of capture and time of bleeding were recorded, and the time elapsed between the two (hereafter “bleed time”) was used to assess potential effects of time until bleeding on metabolite profiles. During spring, we also collected toenails of dunlins which can be used as a tool to identify the non-breeding origin of dunlins (see [43] and “Data analysis”). Between 1 and 2 mm of toenail were clipped from three to four toes of each bird, using sharp scissors, and stored in individual plastic bags.

This study was carried out in strict accordance with the recommendations of the Animal Welfare Body (ORBEA) of the Faculty of Sciences of the University of Lisbon which also approved the research protocol (Statement 2–2019). Ringing and sampling permits (242/2019) were issued by Instituto da Conservação da Natureza e das Florestas (ICNF).

### Plasma metabolite analysis

Plasma metabolite assays were performed at the “Técnicas Aplicadas a la Biociencia” laboratory (University of Extremadura, Spain) using standard diagnostic kits and a microplate spectrophotometer (SpectraMax Plus 384 system, Molecular Devices, USA). Briefly, total triglycerides (triglycerides plus free glycerol) were measured by a colorimetric assay (No. 10010303, Cayman Chemical); this assay uses the enzymatic hydrolysis of triglycerides by lipase to produce glycerol and free fatty acids. Glycerol concentration was determined by a coupled enzyme assay (No. 10010755, Cayman Chemical); the measurement of circulating glycerol is considered to reflect lipolysis and is also useful for the correction of glycerol interference in measurements of triglycerides. Thus, triglyceride levels were calculated by subtracting free glycerol from total triglyceride level [e.g. 29, 48]. Plasma β-hydroxybutyrate was determined by colorimetric assay kit (No. 700190, Cayman Chemical); measurement of β-hydroxybutyrate provides a reliable index of the level of ketoacidosis (accumulation ketone bodies, mainly from lipid metabolism during fasting). β-hydroxybutyrate determination is based upon the oxidation of D-3-hydroxybutyrate to acetoacetate by the enzyme 3-hydorxybutyrate dehydrogenase. Finally, concentration of uric acid was measured by the colorimetric assay kit (No. 700320, Cayman Chemical), which provides a fluorescence-based method for detecting uric acid in plasma. Eighty percent of all measurements were made in duplicate.

### Leucocyte profiles

Blood smears were air-dried, fixed with absolute metanol, and stained with Hemacolor® Rapid staining kit (Merck KGaA, Germany) and examined using a microscope. A sample of 100 leucocytes was classified into lymphocytes, basophils, heterophils, eosinophils and monocytes [47]. The heterophil/lymphocyte ratio (H/L) was used as an indicator of physiological stress [49].

### Sex determination

DNA was extracted from blood samples (stored in 96% ethanol) with E.Z.N.A Tissue DNA kit (Omega Biotek) following the manufacturer´s instructions. CHD-W and CHD-Z genes were amplified using the 2602F/2669R primers [50]. PCR reactions were carried out in volumes of 10 μL that comprised 5 μL of Qiagen Multiplex PCR Master Mix, 3.2 μL of ultra-pure water, with 0.4 μl of each 10 mM amplification primer and 1 μL of DNA extract. The amplified fragments were separated by capillary electrophoresis on a 2% agarose gel and the males and females were identified by the occurrence of one or two bands, respectively. To confirm the results, the amplification of 47 samples randomly chosen was repeated using P2/P8 primers [51].

### Stable isotope analysis

Toenail and feather (collected from dunlin chicks in Iceland as a proxy for breeding ground origin–see below “Data analysis”) samples were washed in double baths of 0.25 N sodium hydroxide solution alternated with baths of double distilled water to remove any adherent contaminating material [43]. Samples of RBC, plasma and toenails were dried at 50°C for 48 h, grounded to a homogeneous powder and stored (0.6 to 1.0 mg) in tin cups. Stable isotope analysis of carbon and nitrogen was performed at the “Stable Isotopes and Instrumental Analysis Facility” of the Faculty of Sciences, University of Lisbon. δ^13^C and δ^15^N in the samples were determined by continuous flow isotope mass spectrometry (CF-IRMS), on a Sercon Hydra 20–22 (Sercon, UK) stable isotope ratio mass spectrometer, coupled to a EuroEA (EuroVector, Italy) elemental analyser for online sample preparation by Dumas-combustion. δ^15^N_Air_ values are referred to Air and δ^13^C_VPDB_ values are referred to PDB (Pee Dee Belemnite). The reference materials used were USGS-25, USGS-35, BCR-657 and IAEA-CH7; the laboratory standard used was Rice Flour. Uncertainty of the isotope ratio analysis, calculated using values from 6 to 9 replicates of laboratory standard interspersed among samples in every batch of analysis, was ≤ 0.1‰.

### Prey availability and foraging performance of dunlins

Prey availability and foraging performance of dunlins were assessed at the Tagus estuary during autumn migration (2019) following the fieldwork and laboratory protocols described in Martins et al. [52]. We performed a between-season comparison, using our data and that published by Martins et al. [52] for the spring season at the same study area. Briefly, prey density was determined through the analysis of sediment cores for all macroinvertebrates except for shrimps that were sampled with a landing net. Invertebrate biomass (AFDW/m^2^) was estimated from different measurements of intact structures in individuals found in cores and using published relationships between size and biomass for each species (see detailed methodological protocols in S1 File and S1 Table).

To determine feeding performance (prey consumed and intake rates) of dunlins, a total of 107 video recordings of foraging birds with approximately one minute of duration were performed during diurnal low tides (see S1 File for detailed methodological protocols).

### Data analysis

During spring, passage migrant dunlins from Mauritania were identified by their typical (high) δ^13^C and (low) δ^15^N ratios in toenails, following Catry et al. [43, 44] (see S1 Fig). Only these birds were included in the subsequent analyses. These represent the bulk of migrant birds using the Tagus estuary as a stopover [43, 44]. We employed isotopic clock models to determine time since arrival (TSA; corresponding to the time since diet shift) of individuals at the Tagus estuary from signatures in RBC and plasma (see complete methodology in [40]). Very briefly, the method uses the values of typical (reference) isotopic values of birds at their origin and at their destination to derive the TSA of an individual bird estimated by the magnitude of the deviation of its isotopic signature from the origin, towards the reference values at its destination. This deviation is estimated assuming that the isotopic signature after a dietary shift follows an exponential function [40]. The mean coefficient of variation of the TSA estimates (calculated from 1000 replications for each individual [40]) was 22%. After estimating TSA, we calculated date of arrival at the Tagus estuary by subtracting TSA from the day of capture event. We then pooled date of arrival in three stages (1) “early” (from 28 March to 9 April), (2) “mid” (from 14 April to 4 May) and (3) “late” (from 5 May to 14 May). Stages were defined based on the distribution of arrival dates of sampled birds, considering that “early” and “late” migrants correspond to the first and third quantiles. For autumn migration, capture date was used as a proxy for time of migration, as we were unable to estimate TSA from stable isotopes [40]. Given that capture events were spaced out by ca. 2 weeks, if stationary periods are shorter than that (as in spring [40]), capture date can indeed be used to distinguish between early (18 July), mid (1 August) and late (16 August) migrants.

In order to interpret migratory strategies of autumn migrant dunlins, namely to assess the potential of a direct flight between the most likely breeding area (Iceland) and the Tagus estuary, carbon and nitrogen isotopic signatures recorded in RBC of autumn migrants were compared to the expected reference values of both sites. We used body feathers of dunlin chicks collected in Iceland (n = 8) as a proxy for the breeding ground signature and RBC of overwintering dunlins (birds captured in spring with toenail δ^13^C and δ^15^N ratios typical from the Tagus estuary, n = 7; see [43, 44]) as a proxy for the Tagus estuary signature.

Scaled mass index (SMI), as a proxy for body condition of dunlins, was calculated as follow:

$${\hat{M}}_{i}=M_{i}{\left[\frac{L_{0}}{L_{i}}\right]}^{b_{SMA}}$$

where *M*_*i*_ and *L*_*i*_ are the body mass and the wing length of individual *i* respectively; bSMA is the scaling exponent estimated by the standardised major axis (SMA) regression of M on L; L_0_ is the mean value of wing length of the sampled population; and ${\hat{M}}_{i}$ is the predicted body mass for individual *i* when the wing length is standardized to *L*_*0*_ (see [53] for further details). We estimated SMI for males and females separately, as they are sexually dimorphic.

We run generalized linear models (GLMs) to investigate variation in (log-transformed) plasma metabolite concentrations across and between migratory periods. We included plasma metabolite concentrations as the response variable (separate single response models were run for each metabolite), stage (i.e. early, mid and late migrants for within season comparisons) or migration period (spring vs autumn for seasonal comparisons) and sex as fixed factors, and bleed time, SMI and TSA (for spring migration) as covariates. The interactions between stage or migration period and SMI, and the interaction between stage and TSA (in spring) were also included in the full model. Since we estimated SMI for males and females separately (therefore already accounting for the potential impact of sex on body condition of dunlins), and we do not expect the impact of bleed time on metabolite concentration to vary between sexes, sex was not included in any interaction. Model selection was performed by comparing increasingly simple nested models, successfully removing non-significant predictors and interactions, with likelihood ratio tests (F-tests). Results are presented for the minimum adequate model.

GLMs and the same model selection procedures were used to investigate relationships between metabolite concentrations and TSA (in spring) and fat scores, including bleed time and SMI as covariates and sex as fixed factor. One-way ANOVAs were used to assess differences in haematological parameters between and within seasons, using log-transformed data whenever necessary to ensure normal distribution of data. Holm-Bonferroni corrections were applied to account for familywise (Type I) error rates in multiple comparisons performed to compare haematological parameters between migration periods and within each migratory period.

All analyses were run using R software [54], version R-4.1.1.

## Results

Overall, we captured 77 and 58 dunlins at the Tagus estuary during spring and autumn seasons, respectively. Following the discriminant analysis protocol proposed by Catry et al. [43, 44] based on the stable isotope signatures of toenails, 65 of the birds captured during spring were assigned to the wintering population of Mauritania, seven were overwintering birds from the Tagus estuary, and five could not be assigned to any of these two populations (total assignment rate = 93.5%; S1 Fig).

### Variation in plasma metabolite profiles within and between migratory periods

Triglycerides were positively correlated with glycerol and negatively correlated with β-hydroxybutyrate concentrations (R^2^ = 0.346, p<0.001 and R^2^ = 0.045, p = 0.012, respectively).

**Fig 1:**
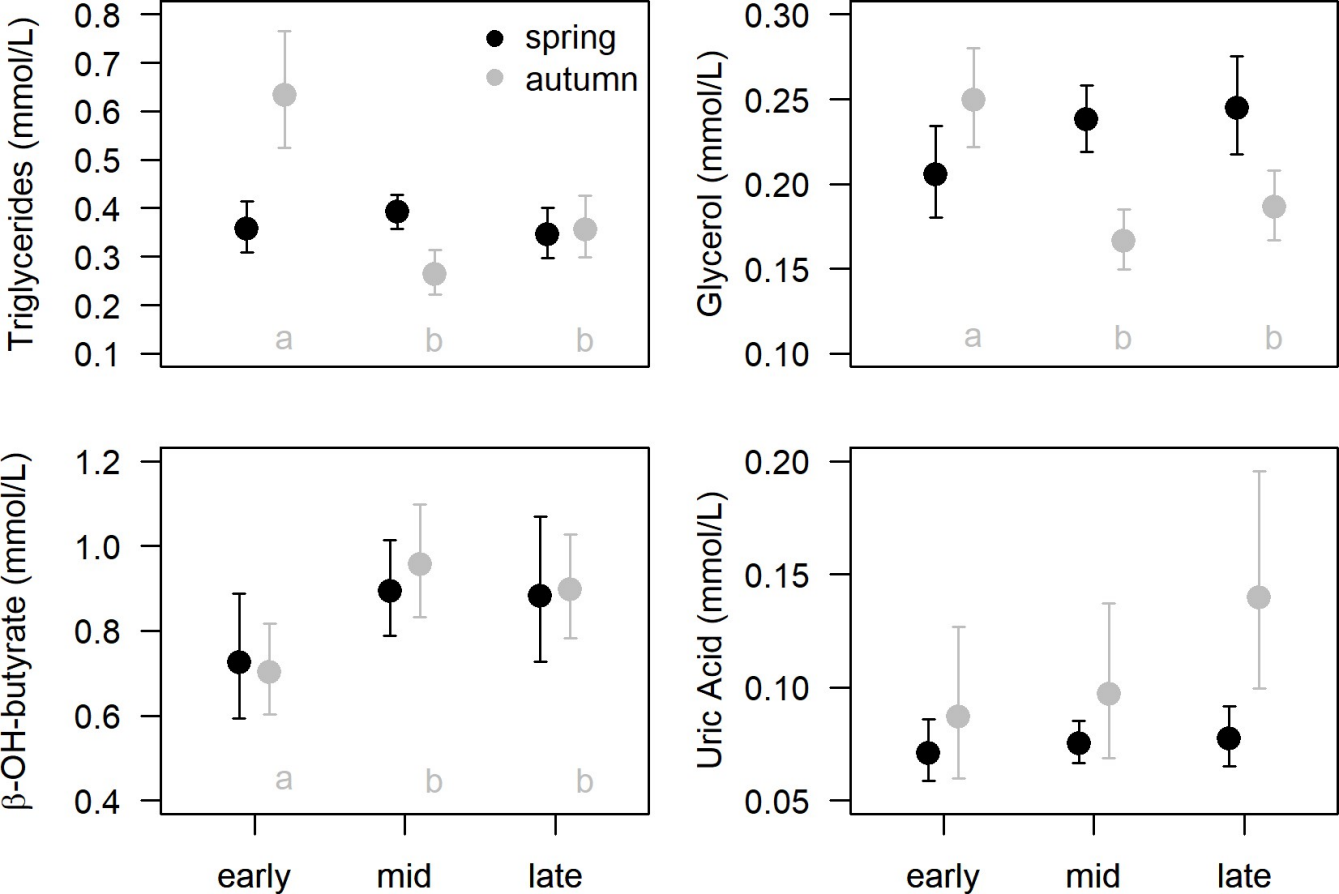
Plasma metabolite concentrations of dunlins sampled at the Tagus estuary at different migratory stages (early, mid and late) during spring and autumn migrations. Data (mean ± confidence intervals of the mean) are predictions from the selected models (see Table 1) where significant covariates were set with the same mean value in all groups. Different letters indicate significant differences between stages within the same migration (see Results). N = 13, 33 and 16 for early, mid and late stages, respectively, in spring migration; N = 16, 19 and 18 (20 for β-hydroxybutyrate and uric acid) for early, mid and late stages, respectively, in autumn migration.

Plasma metabolite concentrations did not differ significantly among early, mid and late migrants during spring migration (Table 1, Fig 1). In this period, birds with higher SMI had higher triglycerides and lower β-hydroxybutyrate concentrations, after correcting bleed time (triglycerides decreased with increasing bleed time; Table 1). In autumn, early migrants had higher values of triglycerides than mid and late migrants, and higher glycerol concentrations than mid migrants. The opposite pattern was found for β-hydroxybutyrate, with concentrations of this metabolite increasing later in the season (Table 1, Fig 1). β-hydroxybutyrate levels significantly decreased with SMI in autumn (Table 1).

**Table 1:** Results of the minimum adequate model derived by comparison of nested generalized linear models explaining variation in plasma metabolite concentrations of migrant dunlins across stages (early, mid and late) in spring and autumn migratory periods.

		Intercept	Stage (early)	Bleed time	Scaled mass index (SMI)
**SPRING**	**Triglycerides**	-1.310 ± 0.265	mid: 0.090 ± 0.083 (p = 0.284)	-0.002 ± 0.001 **(p<0.001)**	0.012 ± 0.005 **(p = 0.020)**
			late: -0.036 ± 0.114 (p = 0.751)		
	**Glycerol**	-1.582 ± 0.066	mid: 0.146 ± 0.077 (p = 0.063)		
			late: 0.175 ± 0.088 (p = 0.052)		
	**β-OH-butyrate**	0.892 ± 0.338	mid: 0.208 ± 0.117 (p = 0.081)		-0.025 ± 0.007 **(p<0.001)**
			late: 0.195 ± 0.143 (p = 0.177)		
	**Uric Acid**	-2.646 ± 0.095	mid: 0.060 ± 0.113 (p = 0.596)		
			late: 0.085 ± 0.128 (p = 0.510)		
**AUTUMN**	**Triglycerides**	-0.456 ± 0.094	mid: -0.879 ± 0.128 **(p<0.001)**		
			late: -0.576 ± 0.129 **(p<0.001)**		
	**Glycerol**	-1.389 ± 0.058	mid: -0.405 ± 0.079 **(p<0.001)**		
			late: -0.291 ± 0.080 **(p<0.001)**		
	**β-OH-butyrate**	0.889 ± 0.389	mid: 0.309 ± 0.103 **(p = 0.004)**		-0.027 ± 0.008 **(p = 0.002)**
			late: 0.245 ± 0.102 **(p = 0.019)**		
	**Uric Acid**	-2.442 ± 0.188	mid: 0.109± 0.255 (p = 0.671)		
			late: 0.473 ± 0.252 (p = 0.066)		

The regression coefficients ± standard deviation and associated p-values are presented for the variables retained in each final model.

**Fig 2:**
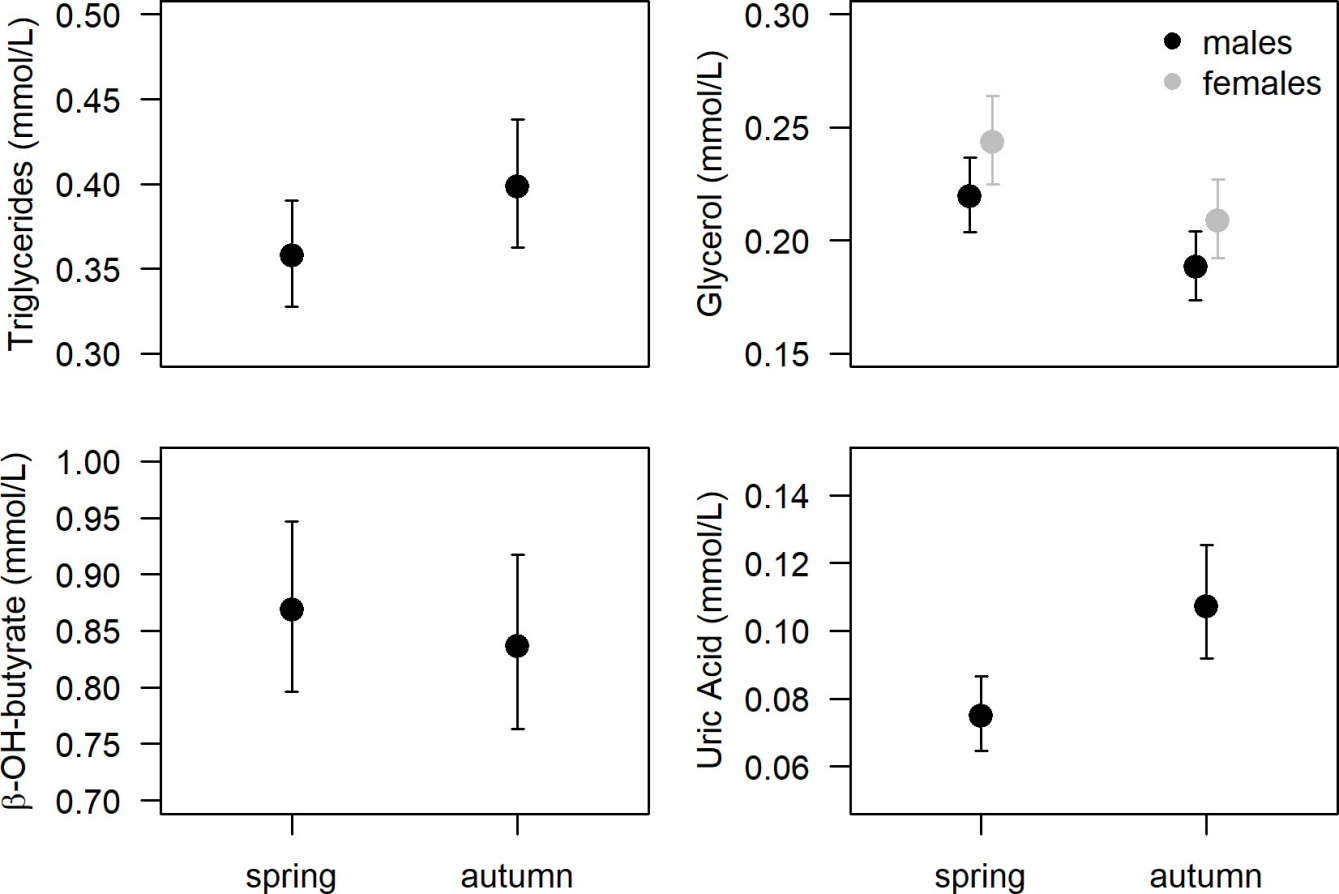
Plasma metabolite concentrations of dunlins sampled at the Tagus estuary during spring (n = 62) and autumn (n = 53–55) migration. Data (mean ± confidence intervals of the mean) are predictions from the selected model (see Table 2) where significant covariates were set with a constant mean value. For glycerol, predictions are presented for males and females separately, as sex was retained in the final model. Glycerol and uric acid concentrations were significantly higher and lower in spring than in autumn migrants, respectively (see Results).

Between-season comparisons yielded no significant differences on triglycerides and β-hydroxybutyrate concentrations between spring and autumn migrants; however, glycerol levels were higher in spring and uric acid was higher in autumn migrants (Table 2, Fig 2).

**Table 2:** Results of the minimum adequate model derived by comparison of nested generalized linear models explaining variation in plasma metabolite concentrations of migrant dunlins between migratory seasons.

	Intercept	Migration (autumn)	Bleed time	Scaled mass index (SMI)	Sex (male)
**Triglycerides**	-1.087 ± 0.257	-0.108 ± 0.066 (p = 0.104)	-0.003 ± 0.0004 **(p<0.001)**	0.011 ± 0.005 **(p = 0.039)**	
**Glycerol**	-1.454 ± 0.059	0.154 ± 0.047 **(p = 0.001)**	-0.001 ± 0.0003 **(p = 0.004)**		-0.104 ± 0.047 **(p = 0.028)**
**β-OH-butyrate**	0.993 ± 0.242	0.037 ± 0.064 (p = 0.562)		-0.025 ± 0.005 **(p<0.001)**	
**Uric Acid**	-2.233 ± 0.078	-0.360 ± 0.108 **(p = 0.001)**			

The regression coefficients ± standard deviation and associated p-values are presented for the variables retained in each final model.

### Fat scores and plasma metabolites

During spring, fat scores of migrant dunlins increased significantly with TSA (regardless of migration stage and sex; β = 0.170 ± 0.025, p<0.001) and mid and late migrants showed higher fat loads than early ones (mid vs early β = 0.853 ± 0.367, p = 0.024; late vs. early β = 2.774 ± 0.422, p<0.001). In autumn, no significant differences were found on fat scores among dunlins from different migratory stages (regardless of sex; mid vs early β = -0.520 ± 0.316, p = 0.103; late vs. early β = -0.463 ± 0.338, p = 0.175). Overall, no significant differences were recorded in fat scores between spring and autumn migrants (F_1, 121_ = 0.792, p = 0.375).

Concentrations of triglycerides were higher in birds with larger fat scores in both spring and autumn (Table 3). The same positive trend was recorded for glycerol in spring, whereas the opposite pattern was documented for β-hydroxybutyrate and uric acid in autumn (Table 3). During spring migration, triglycerides levels were positively correlated to TSA (Table 4).

**Table 3:** Results of the minimum adequate model derived by comparison of nested generalized linear models explaining the relationships between plasma metabolite concentrations and fat scores of migrant dunlins in spring and autumn migratory periods.

		Intercept	Fat	Bleed time	Scaled mass index (SMI)
**SPRING**	**Triglycerides**	-0.978 ± 0.130	0.056 ± 0.022 **(p = 0.013)**	-0.002 ± 0.001 **(p = 0.006)**	
	**Glycerol**	-1.591 ± 0.065	0.038 ± 0.017 **(p = 0.029)**		
	**β-OH-butyrate**	1.038 ± 0.332	0.014 ± 0.032 (p = 0.669)		-0.026 ± 0.008 **(p = 0.002)**
	**Uric acid**	-2.684 ± 0.093	0.026 ± 0.025 (p = 0.299)		
**AUTUMN**	**Triglycerides**	-0.909 ± 0.184	0.098 ± 0.042 **(p = 0.024)**	-0.003 ± 0.001 **(p<0.001)**	
	**Glycerol**	-1.632 ± 0.116	0.040 ± 0.026 (p = 0.142)	-0.001 ± 0.0004 **(p = 0.011)**	
	**β-OH-butyrate**	0.303 ± 0.112	-0.127 ± 0.029 **(p = 0.001)**		
	**Uric acid**	-1.597 ± 0.272	-0.176 ± 0.070 **(p = 0.015)**		

The regression coefficients ± standard deviation and associated p-values are presented for the variables retained in each final model.

**Table 4:** Results of the minimum adequate model derived by comparison of nested generalized linear models explaining the relationships between plasma metabolite concentrations and time since arrival (TSA) of migrant dunlins in spring.

	Intercept	Time since arrival (TSA)	Bleed time	Scaled mass index (SMI)
**Triglycerides**	-0.764 ± 0.076	0.011 ± 0.005 **(p = 0.040)**	-0.002 ± 0.001 **(p<0.001)**	
**Glycerol**	-1.498 ± 0.045	0.007 ± 0.005 (p = 0.234)		
**β-OH-butyrate**	1.065 ± 0.323	0.009 ± 0.009 (p = 0.318)		-0.027 ± 0.007 **(p<0.001)**
**Uric acid**	-2.605 ± 0.064	0.002 ± 0.007 (p = 0.794)		

The regression coefficients ± standard deviation and associated p-values are presented for the variables retained in each final model.

### Variation in haematological profiles within and between migratory periods

**Fig 3:**
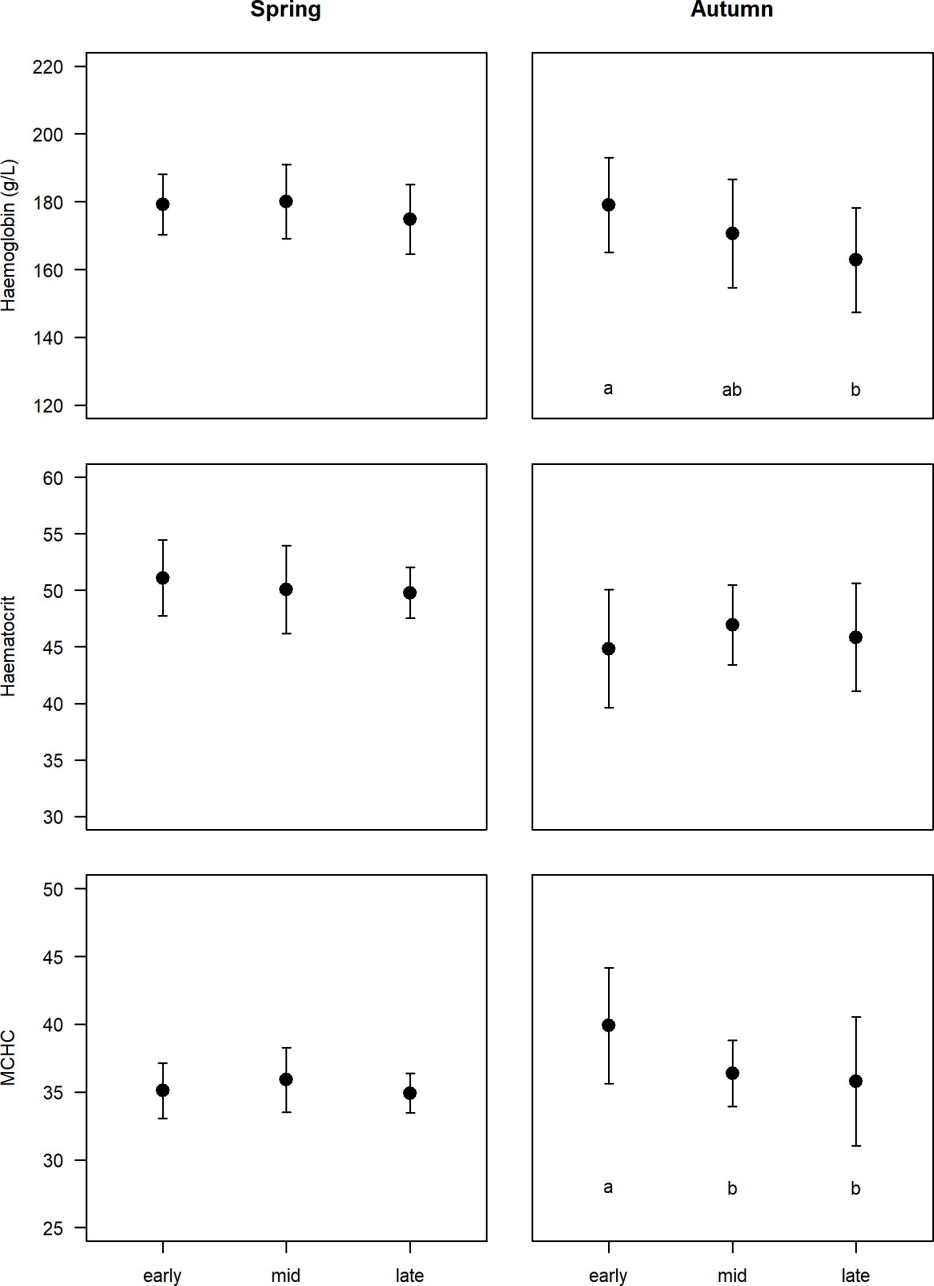
Haemoglobin (Hb), haematocrit and mean cell haemoglobin concentrations (MCHC) in the blood of dunlins during different stages (early, mid, late) of spring and autumn migration. Different letters indicate significant differences between stages (see Results).

**Fig 4:**
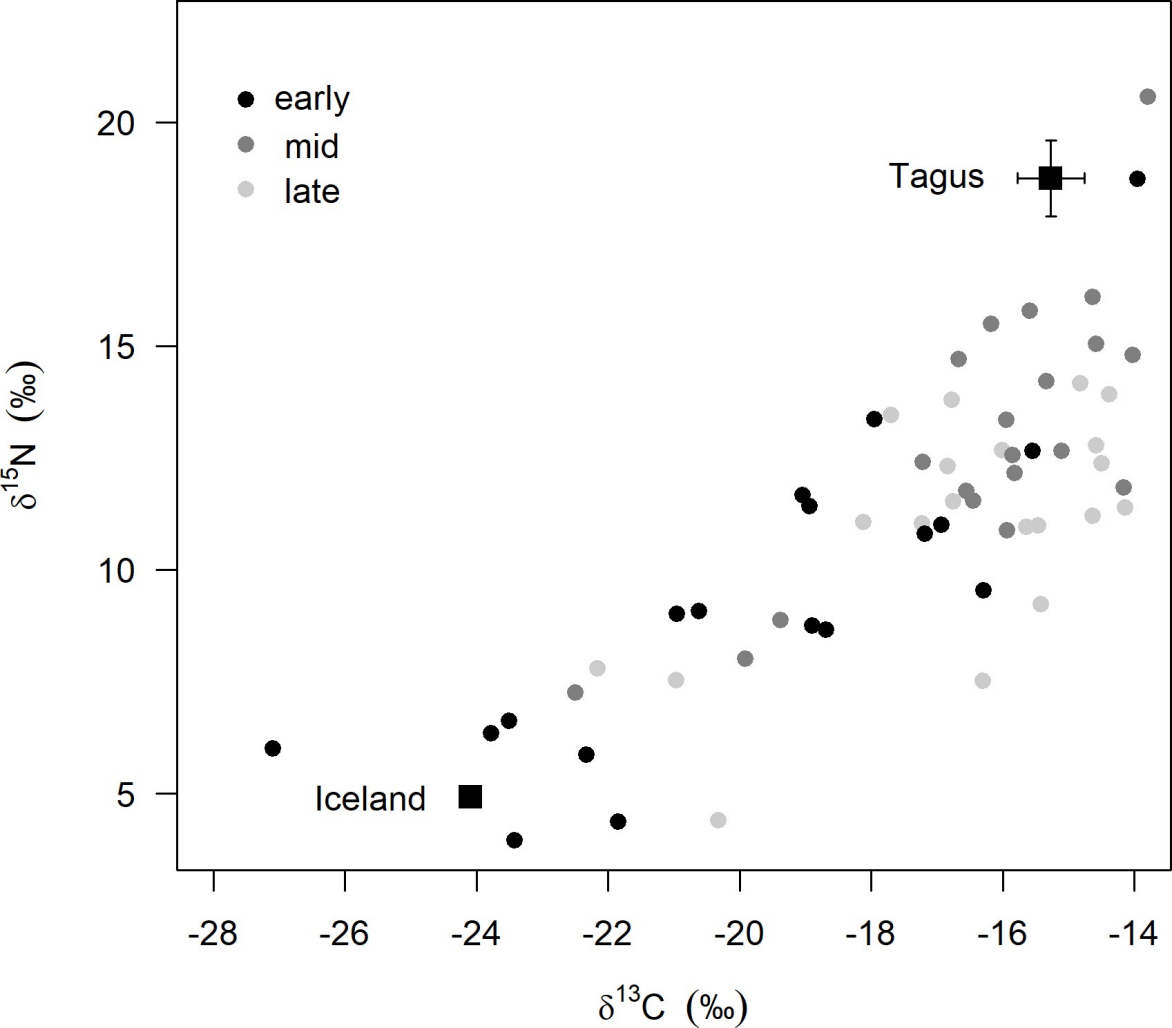
Carbon and nitrogen stable isotope signatures in red blood cells of dunlins sampled at the Tagus estuary during autumn migration. Early, mid and late correspond to three capture events (mid-July, early August and mid-August). Square symbols represent the expected signatures of dunlins (mean ± SD) at Iceland (obtained from chick feathers, n = 8) and Tagus estuary (obtained from overwintering birds during spring, n = 7).

Haemoglobin concentrations, haematocrit and MCHC did not differ between male and female dunlins (F_1,118_ = 2.366, p = 0.127, F_1,102_ = 0.528, p = 0.469 and F_1,102_ = 0.147, p = 0.702, respectively). During spring, haemoglobin, haematocrit and MCHC levels did not vary significantly among stages (F_2,60_ = 1.34, p = 0.27, F_2,47_ = 0.529, p = 0.593 and F_2,47_ = 1.164, p = 0.321, respectively, Fig 3) and were unrelated to TSA (r = 0.004, p = 0.608; r = 0.031, p = 0.219 and r = 0.029, p = 0.234, respectively). In autumn, early migrants showed higher values of haemoglobin than late ones (F_2,52_ = 4.902, p = 0.011, post hoc Tukey tests p = 0.008) and had higher MCHC values than mid and late migrants (F_2,49_ = 4.857, p = 0.012, post hoc Tukey tests p = 0.046 and p = 0.011, respectively, Fig 4). No differences were recorded during autumn in haematocrit (F_2,49_ = 0.868, p = 0.426, Fig 3). Overall, haemoglobin concentrations and haematocrit were higher in spring than in autumn (F_1,118_ = 11.76, p<0.001 and F_1,102_ = 26.85, p<0.001), although for haemoglobin the difference seems to lie exclusively on the low values of late autumn migrants. No differences were recorded in MCHC between seasons (F_1,102_ = 4.299, p = 0.041, non-significant after Holm-Bonferroni correction, i.e., assuming α = 0.025).

Leucocyte profiles were similar between spring and autumn migrants (Table 5). During spring, heterophils were higher in early compared to late migrants, lymphocytes were higher in late compared to mid migrants and the H/L ratio was overall lower in late migrants (Table 6). In autumn, early migrants showed lower values of heterophils (Table 6).

**Table 5:** Comparison of leucocyte profiles (mean ± SD) of dunlins between spring and autumn migration.

	Spring	Autumn	ANOVA
**Heterophils**	43.7 ± 15.6	46.2 ± 17.6	F_1,112_ = 0.619; p = 0.433
**Eosinophils**	6.4 ± 5.1	5.6 ± 4.5	F_1,112_ = 0.059; p = 0.808
**Basophils**	5.1 ± 5.5	3.0 ± 4.1	F_1,112_ = 3.608; p = 0.060
**Lymphocytes**	39.0 ± 12.3	38.8 ± 15.3	F_1,112_ = 0.006; p = 0.940
**Monocytes**	5.8 ± 3.8	6.3 ± 4.0	F_1,112_ = 0.614; p = 0.435
**Polychromatophilic erythrocytes**	15.9 ± 9.4	12.9 ± 8.0	F_1,112_ = 3.174; p = 0.078
**H/L**	1.4 ± 1.0	1.6 ± 1.2	F1_,112_ = 2.257; p = 0.136

Data were log-transformed to achieve normal distribution (except for heterophils and lymphocytes).

**Table 6:** Comparison of leucocyte profiles (mean ± SD) among early, mid and late migrant dunlins during spring and autumn migration periods.

	**SPRING**			
	**early**	**mid**	**late**	**ANOVA**
Heterophils	53.4 ± 14.9^a^	44.9 ± 16.0^ab^	35.4 ± 11.3^b^	F_2,55_ = 4.808; **p = 0.012**
Eosinophils	3.7 ± 4.9	6.7 ± 5.0	7.4 ± 5.2	F_2,55_ = 2.194; p = 0.121
Basophils	2.1 ± 1.6	5.9 ± 6.0	5.4 ± 5.6	F_2,55_ = 1.120; p = 0.334
Lymphocytes	35.3 ± 9.5^ab^	36.7 ± 12.8^a^	46.1 ± 10.6^b^	F_2,55_ = 4.012; **p = 0.024**
Monocytes	5.5 ± 3.2	5.8 ± 3.8	5.8 ± 4.3	F_2,55_ = 0.007; p = 0.993
Polychromatophilic erythrocytes	12.3 ± 10.0	17.1 ± 9.5	15.6 ± 8.9	F_2,55_ = 2.110; p = 0.131
H/L	1.7 ± 0.9^a^	1.6 ± 1.2^a^	0.8 ± 0.4^b^	F_2,55_ = 4.308; **p = 0.018**
	**AUTUMN**			
	**early**	**mid**	**late**	**ANOVA**
Heterophils	38.4 ± 17.6^a^	54.2 ± 17.9^b^	44.5 ± 14.6^ab^	F_2,53_ = 4.099; **p = 0.022**
Eosinophils	6.8 ± 5.7	4.8 ± 3.0	5.6 ± 4.8	F_2,53_ = 0.416; p = 0.662
Basophils	4.4 ± 6.6	2.3 ± 1.7	2.5 ± 2.9	F_2,53_ = 0.693; p = 0.504
Lymphocytes	44.3 ± 15.2^a^	32.2 ± 16.6^b^	41.1 ± 12.1^ab^	F_2,53_ = 3.390; p = 0.041
Monocytes	6.1 ± 3.6	6.6 ± 3.9	6.4 ± 4.6	F_2,53_ = 0.171; p = 0.843
Polychromatophilic erythrocytes	16.3 ± 9.8	10.6 ± 8.2	12.7 ± 5.1	F_2,53_ = 2.432; p = 0.100
H/L	1.1 ± 0.7^a^	2.3 ± 1.4^b^	1.4 ± 1.2^ab^	F_2,53_ = 3.928; p = 0.026

ANOVAs were followed by post-hoc Tukey HSD tests. Significant differences are highlighted in bold (after adjusting the α-value with the Holm-Bonferroni method for multiple testing, see Methods), and groups sharing the same letter do not differ significantly. Data were log-transformed to achieve normal distribution (except for heterophils and lymphocytes).

### Food availability and foraging performance of dunlins during spring and autumn migration

Overall, the harvestable biomass of macroinvertebrate species that comprise the diet of dunlins at the Tagus estuary was twofold higher in autumn than in spring. Such differences were mostly due to the higher abundance of polychaeta during autumn (S2 Table). Overall, prey consumption and energy intake rates were also higher in autumn, due to the higher consumption of polychaeta (S3 Table).

### Stable isotopic signatures of autumn migrants

Carbon and nitrogen stable isotope signatures in RBC of autumn migrant dunlins in comparison to expected reference values for the breeding area (Iceland) and the Tagus estuary are presented in Fig 4. Early migrants had significantly lower δ^15^N and δ^13^C values than mid and late migrants (One-way ANOVA followed by post-hoc comparisons; F_2,55_ = 6.73, p = 0.002 and F_2,55_ = 10.28, p<0.001, respectively).

Isotopic signatures of autumn migrants were not correlated with individual body condition as measured by SMI (r = 0.018, p = 0.322 and r = 0.008, p = 0.506, respectively for δ^15^N and δ^13^C) and fat scores (r = 1.684e-07, p = 0.998 and r = 0.0001, p = 0.935, respectively for δ^15^N and δ^13^C).

## Discussion

We found evidence for a similar fuelling strategy of spring and autumn migrating dunlins at the Tagus estuary, as revealed by overall similar plasma triglycerides and β-hydroxybutyrate concentrations and haematological profiles (the differences found were not consistent across each season, see below). Martins et al. [52] showed that the Tagus estuary offers above-average feeding conditions for northward migrating dunlins during spring, suggesting that birds can achieve high fattening rates during stopover periods. In comparison to spring, the higher food availability and foraging performance in autumn suggest high-quality conditions for migratory dunlins also in autumn, indicating that potential seasonal differences in refuelling rates may be driven by different migratory strategies rather than food availability. Migrant dunlins arrive at the Tagus estuary generally with medium/high fuel loads and, at least in spring, stay for relatively short periods (7.5 days on average; [40]), suggesting they do not use this site to build very large fat reserves to fuel the next flight. Taken together, these results indicate that dunlins (and possibly other shorebirds) use the Tagus estuary as a stopover rather than a staging site [24]. Our results generally agree with a skipping migratory strategy, consisting of a number of short-to-medium distance bouts with intermittent fuelling along a network of stopover areas [24, 38]. Early autumn migrants showed higher triglycerides and haemoglobin levels, and may therefore follow a slightly different strategy, behaving as jumpers instead of skippers.

### Plasma metabolites as proxies for refuelling rates of migratory dunlins

Overall, triglycerides increased with SMI and fat scores, while the opposite pattern was recorded for β-hydroxybutyrate, confirming that the two metabolites correlate with body mass changes following fast refuelling and slow refuelling/fasting events, respectively [29, 31, 33, 55]. Surprisingly, we found a positive correlation between glycerol and fat scores during spring, while no pattern was evident in autumn. More often, plasma glycerol concentrations increase during periods of fasting and mass loss [33, 55]. However, several studies have found that glycerol may actually increase at very high rates of fat deposition, during rapid fatty acid uptake by adipose tissue and muscle, revealing the complexity in interpreting such patterns [e.g. 29, 31]. The different patterns found in this study do not help clarifying the relationship between this metabolite and fuel deposition rate (but see below). Plasma concentrations of uric acid are related to the protein metabolism and may thus increase during fuelling, following the intake of dietary protein for storage as fat, or during fasting, following the catabolism of body protein [32, 29, 48, 56]. While we found no relationship between uric acid values and SMI in dunlins, previous studies have suggested that potential confounding effects of diet composition may indeed raise difficulty in the interpretation of uric acid values as an indicator of physiological state [27].

### Variation in plasma metabolite and haematological profiles of dunlins between spring and autumn migration

Similar plasma triglycerides and β-hydroxybutyrate concentrations were recorded in dunlins during spring and autumn migration, while glycerol levels were higher in spring and uric acid levels were higher in autumn. The lack of significant differences between seasons in triglycerides and β-hydroxybutyrate concentrations, which are more often shown to correlate with refuelling rates during migratory events [29, 33] suggests a similar fuelling strategy in dunlins using the Tagus estuary while migrating towards their breeding and non-breeding grounds. Nonetheless, the higher levels of glycerol in spring and lower levels of uric acid in autumn are challenging to interpret, also because they are not similarly correlated with SMI or fat scores in both seasons. Moreover, differences found in these two metabolites were not consistent across migration stages, with the differences in glycerol being attributed mainly to the atypical high values in early-autumn migrants and those in uric acid to the higher values of late-autumn migrants. Seaman et al. [27] also found higher levels of glycerol in Western sandpipers (*Calidris mauri*) during northward compared to southward migration, but failed to provide an explanation to such pattern. On the other hand, Gutiérrez et al. [48] recorded the highest uric acid levels in Black-tailed godwits (*Limosa limosa*) during pre-departure stages, suggesting that these values resulted from the breakdown of proteins when birds change body composition while preparing for a migratory flight. However, this hypothesis is not supported by the lower haemoglobin values found in late-autumn migrants (see below).

We found some differences in the haematological profiles of spring and autumn migrants, with the former showing overall higher haemoglobin and haematocrit levels. The differences seem to be mainly driven by the below-average values in these two parameters recorded for late autumn migrants. Previous studies attributed higher values of haemoglobin to higher fat loads carried by late migrants, as these need increased blood oxygen-carrying capacity to cope with carrying considerable fat burdens [57]. At the Tagus estuary, however, fat scores did not differ among autumn sampling events nor between spring and autumn. Haemoglobin was also found to increase in the last days of stopover [37, 57]. However, we have shown that haemoglobin and haematocrit were uncorrelated to TSA in spring, refuting this hypothesis for dunlins at the Tagus estuary, at least during spring. Alternatively, the lower values of these two parameters recorded in late autumn migrants may indicate that our sample potentially included some local overwintering dunlins arriving earlier than expected, rather than supporting the idea of different migratory strategies between spring and autumn migrants.

Overall, leucocyte profiles, a proxy for the immunocompetence and nutritional stress [35, 58], were similar between spring and autumn migrants. Again, this points to similar conditions experienced by dunlins during both migratory paths. The high habitat quality of the Tagus estuary in terms of food supplies in both seasons supports this result.

### Within season variation in plasma metabolite and haematological profiles of migratory dunlins

Migratory events can be more or less protracted, but even in species or populations with high migratory synchrony, migrants at the edges of the schedule range often travel under different selective pressures. Thus, they may show different strategies in order to cope with existing environmental conditions and to avoid compromising key life-cycle events (e.g. breeding) and survival. Such differences are more likely to occur during spring migration, when birds are more constrained by time but also when weather conditions are more unpredictable. Therefore, one could expect that late spring migrants would adopt a time-minimization strategy to maintain time-critical migration schedules. Different strategies for early and late migrants were unveiled in a study with Semipalmated sandpiper (*Calidris pusilla*), where early migrants gained weight at stopover sites, while late migrants travelled with higher fuel loads and used stopovers to rest and maintain fuel loads [29]. Another study suggested that migrating Western sandpipers (*Calidris mauri*) fatten more slowly if they remain longer at stopovers, showing lower triglycerides levels than individuals with short stopovers [28]. In our study we found no differences in triglycerides (or any other metabolite) concentrations in dunlins across the whole spring migratory period. These results suggest that early and late migrants likely share the same strategies, using the Tagus estuary as a stopover in a similar way. In addition, although we found that mid and late migrants have higher fat scores than early ones, and that fat increases with TSA, plasma metabolites were not correlated with fat scores or TSA. Overall, spring migrants also showed similar haematological profiles, with the only difference being a decreasing trend of H/L ratios across the season. Haemoglobin concentration was uncorrelated to TSA, although one could expect higher levels during the last days of stopover [34].

Physiological profiles of autumn migrants showed some variation along the season, with early migrants recording higher levels of triglycerides and, accordingly, lower concentration of β-hydroxybutyrate. Although we found no evidence for differences in fat scores among groups of migrants, SMI was higher in the early migrants. Moreover, haemoglobin and MCHC were also significantly higher in early than in late migrants. Altogether, these results suggest higher refuelling rates and enhanced blood oxygen-carrying capacity in early autumn migrants.

### Linking refuelling rates to migratory strategies

Birds spend up to 90% of the total migration time on feeding and resting activities at stopover sites [7]. While feeding, it is the refuelling rate that, to a great extent, determines the duration of stopover periods, departure fuel load and, ultimately, overall speed of migration [3]. Therefore, stopover duration tends to increase prior to long, energy-demanding flights [3, 7]. The short stopover period of dunlins at the Tagus estuary during spring migration (7.5 days on average, [40]), suggest they can rapidly rebuild their condition after flying from Mauritania (ca. 2200 km) and will probably perform a short trip before the next stop south of the breeding area, as a long flight bout (as the ca. 3350 kms to flight directly to the breeding area) would likely require a longer refuelling period. Previous evidence points for a strong link between Portugal and the British Isles during spring migration ([59], authors’ unpublished data from ringing recaptures), which is in line with our results. Therefore, during spring migration, flying bouts between Mauritania and Iceland are probably all shorter than 2200 km, and birds may be able to complete these flights without having to carry large fat loads or without extensively depleting their initial fuel loads. Baltic dunlins spending the non-breeding season in Mauritania have been shown to complete spring migration using a skipping strategy, with birds performing at least two relatively short stops (6.4 days on average) in either Morocco, Portugal, Spain or France before arriving at the Wadden Sea [19]. Staging at the Wadden Sea averaged 15.9 days but was highly variable. Given that the last bout between the Wadden Sea and the breeding grounds in Finland is relatively short (<2000 kms), longer stays at the Wadden Sea may be explained by an extended preparation for arrival at breeding sites where food may be scarce or waiting for improved weather conditions at high latitudes [19], as described for other species [20, 28, 60]. This strongly suggests that dunlins following a skipping strategy (flying bouts of approximately 2000 kms) do not need long refuelling periods to complete migration. This is supported by evidence from Baltic dunlins performing a long refuelling period (28.1 days) at the Wadden Sea before a long (ca. 4500 km) direct flight to the non-breeding quarters in Mauritania during autumn migration [19].

Although we have no information on stopover duration at the Tagus during autumn, it is rather likely that most autumn migrants also follow a skipping strategy. Again, ringing records seem to support this hypothesis, as most dunlins recovered or marked in Portugal in autumn have been recorded in the British Isles ([59], authors’ unpublished data from ringing recaptures). However, stable isotope signatures of autumn passage dunlins captured at the Tagus estuary suggest that at least some birds (those with more depleted δ^15^N and δ ^13^C values, particularly the earlier migrants) may have arrived directly from their Icelandic breeding grounds. The terrestrial diet of breeding shorebirds leaves a strong isotopic fingerprint that contrasts with the more estuarine and marine diet available in non-breeding (wintering) habitats [61, 62]. Thus, if birds would have stopped in the British Isles (or elsewhere) on their way to the Tagus, and taking into account the intermediate turnover in RBC of dunlins (estimated half-lives of 8.6 days for δ^13^C and 10.2 for δ^15^N; [63]), we would not expect to find such depleted signatures (similar to the known breeding fingerprint) at the Tagus. A direct flight from Iceland represents a journey of approximately 3350 kms, i.e., ca. 30 to 50% longer than a flight from a bird potentially using a stopover site at the northern or southern British Isles, respectively. Remarkably, we found no relationship between isotopic signature and SMI or fat scores of dunlins. These results suggest that the non-stop flight from Iceland to Tagus could still have a relatively low-cost for dunlins or, alternatively, that they leave the breeding grounds with a considerable fat load. Deposition of large fuel loads at the breeding areas prior to migration was recorded for instance in whimbrels (*Numenius phaeopus*) departing from Iceland [20], so it may be possible for dunlins as well. Arriving at the Tagus estuary with some fuel reserves may allow dunlins to stop for relatively short periods (as they do during spring) and fly to their final non-breeding grounds, where they can restore their body condition, without losing much time. Importantly, a rapid southward migration will prevent overlapping moult and migration, a strategy used by most Baltic dunlins [19] but also other shorebirds [e.g. 64, 65]. Indeed, none of the birds sampled for physiological profile was moulting and only 4% (n = 101) of all dunlins captured during the study showed some extent of primary moult. Notwithstanding, early autumn migrants seem to follow a slightly different strategy than mid and late ones, as they show higher refuelling rates. Since this group includes the majority of birds flying directly from Iceland (as suggested by isotopic signatures), then the two occurrences may be linked, and early autumn migrants may indeed be closer to a jumping migratory strategy.

## Conclusions

Our results highlight the role of the Tagus estuary as a high-quality stopover site for intermediate fuelling in dunlins during both spring and autumn migrations. Contrary to our predictions, refuelling rates and haematological profiles of dunlins were overall similar during spring and autumn migrations, providing no support for the hypothesis that time constrains are greater during spring. Dunlins stopping over at the Tagus estuary are likely to follow a skipping migratory strategy, flying short-to-medium distance bouts while fuelling at moderate rates along a network of stopover sites. Early-autumn migrants, however, display higher fuel deposition rates and enhanced blood oxygen-carrying capacity, which suggests they may behave as jumpers rather than skippers. This indicates substantial within-season variation in refuelling performance and highlights the inherent physiological complexity to stopover events.

In a rapidly changing world, where many shorebird populations are declining at steep rates [66, 67], stopover ecology studies are critical to understand the role of particular sites for migratory species, allowing us to identify areas of conservation priority [24, 25, 66]. Among migrants, jumpers and skippers may be particularly vulnerable to habitat loss, as they rely on a small number of key sites to successfully complete migration. Previous studies have identified declines in overwintering shorebird populations at the Tagus estuary, likely due to the loss or degradation of roosting areas [39], and the prospects for the construction of a new international airport will likely reduce the quality of foraging areas [68]. Such context further calls for a deeper understanding of the migratory strategies and stopover ecology of shorebirds at the flyway scale.

## Supporting information

S1 File Methodological protocols to assess prey availability and foraging performance of dunlins during autumn migration. (DOCX)

S1 Table Equations used to calculate biomass (ash free dry weight, AFDW, mg) of dunlin prey items. TL—total length mm; AFDW—ash free dry weight mg; ML—mandible length mm; APL—anterior posterior length; SL—shell length mm.
(DOCX)

S2 Table Seasonal variation (spring vs. autumn migration) in harvestable density and biomass (mean ± SE) of main dunlin prey species at the Tagus estuary. Data were obtained from core samples except for the shrimp *Crangon crangon* (n = 22 square samples; see Methods for further details). All data from spring was obtained from Martins et al. [52].
(DOCX)

S3 Table Consumption rate (prey consumed/min; mean ± SE) and energy intake (J/min;) of main dunlin prey consumed during spring and autumn migration at the Tagus estuary. Energy intake rates in autumn were estimated from one subset of videos (31 from 107) where all prey could be identified to species level. All data from spring was obtained from Martins et al. [52].
(DOCX)

S1 Fig Carbon and nitrogen stable isotope signatures in toenails of dunlins sampled at the Tagus estuary during spring migration. The wintering (non-breeding) origin of dunlins (Mauritania, Tagus estuary and unknown) was assessed following Catry et al. [43, 44].
(DOCX)
