# Supplementary material for: Stopover use of a large estuarine wetland by dunlins during spring and autumn migrations: Linking local refuelling conditions to migratory strategies
Source: PLoS One. 2022 Jan 25;17(1):e0263031. doi: 10.1371/journal.pone.0263031 (PMC8789102; doi:10.1371/journal.pone.0263031)
Supplement: S1 File — (DOCX) [file pone.0263031.s001.docx]

**S1 File. Methodological protocols to assess prey availability and foraging performance of dunlins during autumn migration**

Prey density was determined through the analysis of 60 sediment cores (113.1 cm2) 30 cm deep, randomly collected in three different days in August 2019. The upper 5 cm of each core was sieved through a 0.5 mm mesh, whereas for the remaining sediment we used a 1 mm mesh. Shrimps available at the surface water film were sampled with a styrofoam enclosure (53.5 x44 cm wide and 10 cm high) that was thrown to the sediment surface and its interior area was sieved with a square-shaped landing net with a 1 mm mesh, collecting all shrimps in the sediment surface and water film. This procedure was repeated 20 times. All invertebrates collected were stored in 70 % alcohol and later identified, counted and for a subset of 20 cores invertebrates were also measured in the laboratory using a binocular microscope. Invertebrate biomass (AFDW/m2) was estimated from different measurements of intact structures: the anterior posterior length for bivalves (*Scrobicularia plana*); the shell length for gastropods (Hydrobia ulvae); the mandible length for polychaeta (*Hediste diversicolor*) and the total length for Malacostraca (*Crangon crangon* and *Cyathura carinata*). These measurements were made under a binocular microscope fitted with an eye-piece reticule, to the nearest 0.02 mm. Prey biomass (AFDW/m2) was estimated using published relationships between size and biomass for each species (Table S1). The invertebrates present in the upper 5 cm of core samples (plus shrimps *Crangon crangon*), all polychaeta less than 66 mm in total length and siphons of bivalves longer than 13mm in antero-posterior length of the shell were considered for biomass and density estimations, as these are considered to be harvestable prey to dunlins (Santos et al. 2005, Martins et al. 2013).

To determine feeding performance (prey consumed and intake rates) of dunlins, a total of 107 video recordings of approximately one minute were performed during diurnal low tides (+-2 h from low tide peak) using two cameras (canon POWERSHOT SX70 HS and canon POWERSHOT SX60) at the study area in August 2019. The success of each feeding attempt was recorded, and prey consumed was identified to species level whenever possible, or else categorized as non-identified prey. Energy intake rate was calculated following Martins et al. 2013. The energy content of each prey species was calculated using the median biomass (AFDW) of each prey species found in our core sampling and considered that one gram of AFDW corresponds to approximately 23 Kj. Energy intake rate (J/min) of dunlins was afterwards estimated as the number of prey consumed per minute multiplied by their specific energetic content.
