# Supplementary material for: Stopover use of a large estuarine wetland by dunlins during spring and autumn migrations: Linking local refuelling conditions to migratory strategies
Source: PLoS One. 2022 Jan 25;17(1):e0263031. doi: 10.1371/journal.pone.0263031 (PMC8789102; doi:10.1371/journal.pone.0263031)
Supplement: S1 Table — TL—total length mm; AFDW—ash free dry weight mg; ML—mandible length mm; APL—anterior posterior length; SL—shell length mm. (DOCX) [file pone.0263031.s002.docx]

**S1 Table.** **Equations used to calculate biomass (ash free dry weight, AFDW, mg) of dunlin prey items.** TL - total length mm; AFDW - ash free dry weight mg; ML - mandible length mm; APL - anterior posterior length; SL - shell length mm.

| **Species** | **Equation** | **Source** |
| --- | --- | --- |
| *Hediste diversicolor* | TL = 40.173 × ML-3.4225 | Masero et al. 1999 |
|  | AFDW= 10^(2.53*LOG(TL)-5.94)^ x 0.771× 1000 | Moreira 1995 |
| *Scrobicularia plana* | AFDW= 10^(2.49*LOG(APL)-4.57)^ x 0.795 x 1000 | Moreira 1995 |
| Siphons *Scrobicularia plana* | SLS = 0.9xAPL+1.4  AFDW= SLSx60.00014xAPL^1.69^ | Zwarts et al. 1994 |
| *Hydrobia ulvae* | AFDW= 0.0154 x SL^2.61^ | Santos et al. 2005 |
| *Crangon crangon* | AFDW= 0.2((TL+1.1295) / 4.7906)^3.0725^ | Viegas et al. 2007 |
| *Cyathura carinata* | AFDW= 0.87549 x TL^3.102^ | Cruz et al. 2003 |

**References**

Cruz S, Marques JC, Gamito S, Martins I (2003) Autecology of the Isopod, *Cyathura carinata* (Kroyer, 1847) in the Ria Formosa (Algarve, Portugal). Crustaceana 76: 781-802.

Martins RC, Catry T, Santos CD, Palmeirim JM, Granadeiro JP (2013). Seasonal variations in the diet and foraging behaviour of Dunlins *Calidris alpina* in a South European Estuary: improved feeding conditions for northward migrants. PLoS ONE 8 (12): e81174. doi:10.1371/journal.pone.0081174

Masero JA, Perez-Gonzalez M, Basadre M, Otero-Saavedra M (1999). Food supply for waders (Aves: Charadrii) in an estuarine area in the Bay of Cadiz (SW Iberian Peninsula). Acta Oecologica-International Journal of Ecology 20: 429–434.

Moreira F (1995) Utilização das zonas entre-marés do estuário do Tejo por aves aquáticas e suas implicações para os fluxos de energia na teia trófica estuarina. PhD Thesis; University of Lisbon.

Santos CD, Granadeiro JP, Palmeirim JM (2005). Feeding ecology of Dunlin *Calidris alpina* in a southern European estuary. Ardeola 52: 235–252.

Viegas I, Martinho F, Neto J, Pardal M (2007) Population dynamics, distribution and secondary production of the brown shrimp *Crangon crangon* (L.) in a southern European estuary. Latitudinal variations. Scientia Marina 71: 451–460.
