# Supplementary material for: Stopover use of a large estuarine wetland by dunlins during spring and autumn migrations: Linking local refuelling conditions to migratory strategies
Source: PLoS One. 2022 Jan 25;17(1):e0263031. doi: 10.1371/journal.pone.0263031 (PMC8789102; doi:10.1371/journal.pone.0263031)
Supplement: S2 Table — Data were obtained from core samples except for the shrimp Crangon crangon (n = 22 square samples; see Methods for further details). All data from spring was obtained from Martins et al. [52]. (DOCX) [file pone.0263031.s003.docx]

**S2 Table. Seasonal variation (spring vs. autumn migration) in harvestable density and biomass (mean ± SE) of main dunlin prey species at the Tagus estuary.** Data were obtained from core samples except for the shrimp *Crangon crangon* (n=22 square samples; see methods for further details). All data from spring was obtained from Martins et al. 2013.

|  | **Density (inds/m^2^)** | | **Biomass (g of AFDW/m^2^)** | |
| --- | --- | --- | --- | --- |
|  | **Spring**  **(n=114 cores)** | **Autumn**  **(n=60 cores)** | **Spring**  **(n=114 cores)** | **Autumn**  **(n=20 cores)** |
| *Cyathura carinata* | --- | 20.63 ± 6.08 | --- | 0.02 ± 0.01 |
| *Hediste diversicolor* | 381 ± 31 | 873.86 ± 57.21 | 3.34 ± 0.27 | 9.90 ± 0.59 |
| *Hydrobia ulvae* | 3768 ± 263 | 358.09 ± 47.06 | 0.27 ± 0.02 | 0.12 ± 0.03 |
| *Nephtys hombergii* | --- | 35.37 ± 8.72 | --- | --- |
| *Scrobicularia plana* | 304 ± 41 | 212.20 ± 23.89 | 0.49 ± 0.07 | 0.15 ± 0.05 |
| *siphons Scrobicularia plana* | 181.6 ± 11.7 | 139.99 ± 11.76 | 0.43 ± 0.03 | 0.11 ± 0.02 |
| *Crangon crangon* | 11.5 ± 5.4 | 23.8 ± 3.5 | 0.05 ± 0.01 | 0.04 ± 0.00 |
| **Total biomass** |  |  | **4.58** | **10.75** |
