# Supplementary material for: Stopover use of a large estuarine wetland by dunlins during spring and autumn migrations: Linking local refuelling conditions to migratory strategies
Source: PLoS One. 2022 Jan 25;17(1):e0263031. doi: 10.1371/journal.pone.0263031 (PMC8789102; doi:10.1371/journal.pone.0263031)
Supplement: S3 Table — Energy intake rates in autumn were estimated from one subset of videos (31 from 107) where all prey could be identified to species level. All data from spring was obtained from Martins et al. [52]. (DOCX) [file pone.0263031.s004.docx]

**S3 Table.** **Consumption rate (prey consumed/min; mean ± SE) and energy intake (J/min;) of main dunlin prey consumed during spring and autumn migration at the Tagus estuary.** Energy intake rates in autumn were estimated from one subset of videos (31 from 107) where all prey could be identified to species level. All data from spring was obtained from Martins et al. 2013.

| **Prey** | **Spring (n=56)*** | | **Autumn (n=107 & n=31)** | |
| --- | --- | --- | --- | --- |
|  | **Consumption rate (prey/min)** | **Energy intake (J/min)** | **Consumption rate (prey/min)** | **Energy intake (J/min)** |
| *Scrobicularia plana* | 0.3 | 16.3 | 0.17 ± 0.05 | 18.2 ± 2.87 |
| *Hydrobia ulvae* | NA | 2.3 | 1.04 ± 0.14 | 13.0 ± 10.5 |
| *Hediste diversicolor* | 3.3 | 230.2 | 4.87 ± 0.36 | 1188.4 ± 80.7 |
| *Crangon crangon* | 0.5 | 66.3 | 0.11 ± 0.04 | 34.6 ± 8.7 |
| Siphons  *Scrobicularia plana* | 0.9 | 41.9 | 0.74 ± 0.09 | 21.9 ± 1.8 |
| Non-identified | - | - | 1.93 ± 0.17 | - |
