# Supplementary material for: Stopover use of a large estuarine wetland by dunlins during spring and autumn migrations: Linking local refuelling conditions to migratory strategies
Source: PLoS One. 2022 Jan 25;17(1):e0263031. doi: 10.1371/journal.pone.0263031 (PMC8789102; doi:10.1371/journal.pone.0263031)
Supplement: S1 Fig — The wintering (non-breeding) origin of dunlins (Mauritania, Tagus estuary and unknown) was assessed following Catry et al. [43, 44]. (DOCX) [file pone.0263031.s005.docx]

**S1 Fig.** **Carbon and nitrogen stable isotope signatures in toenails of dunlins sampled at the Tagus estuary during spring migration.** The wintering (non-breeding) origin of dunlins (Mauritania, Tagus estuary and unknown) was assessed following Catry et al. [43, 44].**
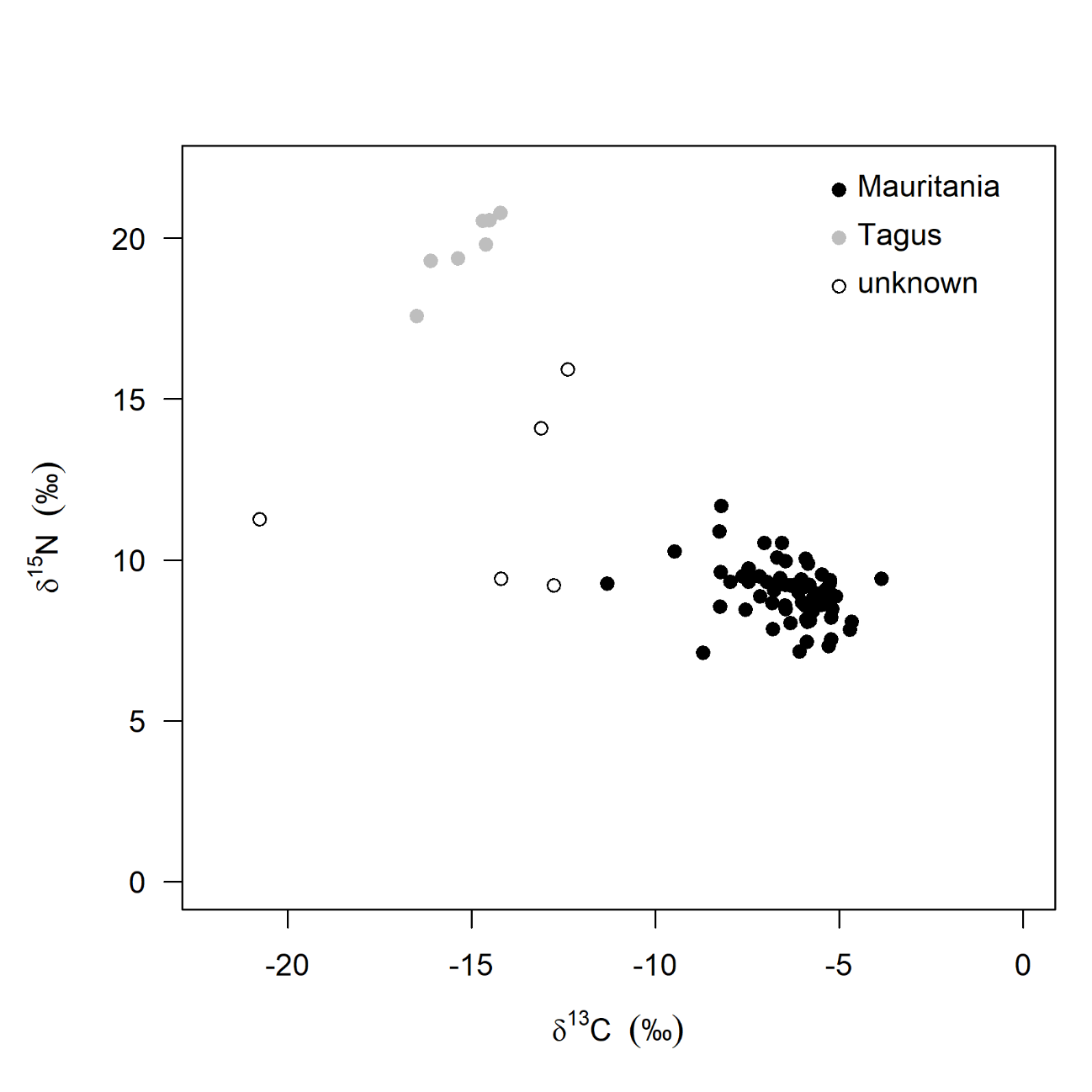
**
